# Enhancing Engagement of Fathers in Web-Based Preventive Parenting Programs for Adolescent Mental Health: A Discrete Choice Experiment

**DOI:** 10.3390/ijerph182312340

**Published:** 2021-11-24

**Authors:** Ashlyn Hansen, Scott D. Brown, Marie B. H. Yap

**Affiliations:** 1 Turner Institute for Brain and Mental Health, School of Psychological Sciences, Monash University, Clayton, VIC 3800, Australia; ashlyn.hansen@monash.edu; 2 School of Psychology, University of Newcastle, Callaghan, NSW 2308, Australia; scott.brown@newcastle.edu.au; 3 Melbourne School of Population and Global Health, University of Melbourne, Melbourne, VIC 3000, Australia

**Keywords:** adolescent, fathers, mental health, parenting, prevention, discrete choice experiment

## Abstract

Few fathers enrol in web-based preventive parenting programs for adolescent mental health, despite the evidence of the benefits associated with their participation. To inform the development of father-inclusive programs, this study used a discrete choice experiment (DCE) design to determine (a) the relative influence of number of sessions, program benefits, program participants, and user control over program content on fathers’ preferences for web-based preventive parenting programs; and (b) whether selected father characteristics were associated with their preferences. One hundred and seventy-one fathers completed the DCE survey, which comprised 25 choices between hypothetical programs. Programs that included the participant’s adolescent child (*z* = 10.06, *p* < 0.0001), or parenting partner (*z* = 7.30, *p* < 0.001) were preferred over those designed for fathers only. Participants also preferred program content that was recommended for them by experts (*z* = −4.31, *p* < 0.0001) and programs with fewer sessions (*z* = −2.94, *p* < 0.01). Program benefits did not predict fathers’ choice of program. Prior use of a parenting program, level of education, perceived role of parenting for adolescent mental health, and being part of a dual-working family were associated with preferences. Application of these findings may improve paternal enrolment in web-based preventive parenting programs.

## 1. Introduction

Mental health problems are one of the leading causes of disability and disadvantage for adolescents worldwide [1,2]. Estimates place global prevalence of mental disorders in children and adolescents between 10–20% [3]. Early-onset mental disorders are associated with adverse lifetime outcomes including impaired academic achievement, unemployment, poor social functioning, and substance use problems [4,5]. Adolescence describes the developmental stage following the onset of puberty, during which young people transition from childhood into adulthood. This period is associated with psychological development and shifts in social environments which can have an impact on mental health and well-being [6]. This includes a transition towards individuation from the family system, increased importance of peer group relations, and personal identity formation. Consequently, young people are at greater risk of experiencing mental health problems during adolescence compared to childhood [6,7]. Given that adolescence is a period of development associated with heightened risks for mental health problems and the onset of many psychiatric illnesses [8], it also presents a key opportunity for preventive intervention.

In the interest of reducing associated social, economic and health costs, evidence-based interventions have been developed to address factors that affect an adolescent’s risk of mental health problems. Meta-analytic reviews have shown that various parenting behaviors, such as harsh parenting and monitoring, are important risk or protective factors for adolescent mental health [9,10,11]. Thus, preventive parenting programs seek to improve adolescent mental health outcomes by increasing parents’ skills and confidence, and by reducing barriers to effective parenting. Such programs have been demonstrated to successfully and cost-effectively reduce the risk and subsequent burden of adolescent mental health problems, with effects lasting up to 15 years post-intervention [12,13,14,15].

Despite the promise of preventive parenting programs, poor parental engagement presents a significant barrier to effectively disseminating these interventions at the scale required to effect population-level benefits for adolescent mental health. Uptake of parenting programs is often as low as 20–30% of target populations [16,17], and parents who experience greater adversity such as single parenthood, a lower socio-economic position, and more stressful life events are frequently under-represented amongst users of preventive parenting programs [18]. In order to optimise the population-level benefits of preventive parenting programs, greater efforts are needed to increase accessibility for diverse parent groups who are less likely to enrol in parenting programs. Fathers and other male caregivers are one group that have been consistently shown to enrol in parenting programs at lower rates than female caregivers [19,20,21]. Although there is limited conclusive research into causal factors for fathers’ low participation rates in preventive parenting interventions, some proposed factors include lack of knowledge and awareness of parenting interventions, masculine beliefs about help-seeking for mental health and parenting, maternal ‘gatekeeping’ (mothers’ beliefs and behaviors that prohibit or facilitate collaborative efforts with fathers in child-rearing) [22], and pragmatic barriers such as fitting attendance around work commitments [23,24,25]. Notably, as fathers participate in program development and evaluation research at lower rates than mothers, it has also been posited that program content may fail to meet fathers’ unique needs and parenting priorities [19,20].

This is particularly concerning given evidence of fathers’ unique and important role in the aetiology and prevention of adolescent mental health conditions [26,27]. Although fathers are under-represented within the parenting literature relative to mothers, comparisons show that paternal parenting factors are as strongly associated with adolescent mental health outcomes as maternal parenting factors [10,28]. Longitudinal evidence also suggests that father involvement in their child’s life protects against psychological distress across the lifespan, independently from maternal involvement [29]. Given the need to improve father engagement in parenting interventions, web-based parenting programs have emerged as a promising alternative which offer flexibility, accessibility, and greater potential for individualisation compared to face-to-face programs [30]. It has been suggested that the accessibility and capacity for individualisation of web-based parenting programs may mean they hold greater appeal for fathers [31]. In a 2017 Australian survey fathers rated internet-based parenting programs as their most preferred delivery format, over one-off or weekly face-to-face sessions [25]. Nevertheless, emerging evidence indicates that male parents are less likely than female parents to access parenting resources via the internet [32], and there has been limited research into how web-based parenting programs can be optimized to better engage fathers of adolescents. Developing a clearer understanding of fathers’ preferences for program delivery is a priority for increasing their uptake of web-based parenting programs. Discrete choice experiments (DCEs) are one approach that may facilitate a better understanding of fathers’ decision-making regarding engagement in web-based parenting programs.

DCEs are a methodology which allow researchers to determine the relative strength of predictors of consumer choices using stated preference data. Participants are presented with a series of choices between alternative products, which are systematically varied on specified attributes (in the case of this study, features of web-based preventive parenting programs, which will be detailed in the DCE development section of this paper). It is assumed that individuals make decisions based on the principle of utility maximisation. In other words, when faced with a choice between products, individuals will typically select the alternative which affords them the greatest value or benefit. This value or utility is assumed to derive from the constituent characteristics (‘attributes’) of a product or type of healthcare service. Therefore, utility can be divided into systematic (derived from explainable attributes and covariates) and random components (comprising all remaining, unidentifiable factors). Discrete choice models thus describe changes in the likelihood a consumer selects a particular product or type of healthcare service, corresponding to manipulation of their properties (or attribute levels) and covariates. DCEs have been increasingly utilized in health economics to address key policy issues, as they can provide insight into consumer preferences to improve adherence to public health programs, and can be used to quantify trade-offs consumers and other agents in the health sector are willing to make between different aspects of programs [33].

### 1.1. The Current Study: Selecting Attributes for the Discrete Choice Experiment

In the current study, we examined the influence of selected program attributes on fathers’ preferences for web-based preventive parenting programs using a DCE design. Design of DCEs requires the development of attribute sets which are salient, plausible, and capable of being traded [34], and include a manageable number of attributes so that participant cognitive burden is minimized [35]. Selected attributes should be able to inform realistic modifications or design features that can be implemented by program developers to improve their appeal for fathers. The paucity of research exploring reasons for fathers’ under-engagement with preventive parenting interventions adds complexity to attribute development. Louviere et al. [36] and Helter and Boheler [35] recommend supplementing literature reviews with qualitative data to inform DCE development, particularly in areas where existing evidence is scant. The methods section of this paper details how we used qualitative methods [37] to supplement a literature review to inform the selection of attributes that inform fathers’ engagement with web-based preventing parenting programs for adolescent mental health. Nevertheless, we will present here a brief review of the literature supporting the attributes and associated hypotheses which were included in the present study.

### 1.2. Number of Sessions

Number of sessions describes the amount of time required to complete a program in full, operationalized as the number of sessions (or modules) in the program. Cunningham et al. [38] found that fathers were most likely to belong to an ‘Information’ oriented segment of parents who were highly influenced by the time demand associated with brief informational resources provided to families waitlisted for child mental health services. A smaller time requirement was preferred by this segment, in contrast to the preferences of other parent segments. Participants in this study were parents of children already experiencing mental health problems, and just over 20% were fathers. Given the small proportion of fathers, it is likely that those who self-selected to participate in the study had exceptionally great interest in, and motivation for, using child mental health resources. A preference for brief programs may be even more strongly pronounced amongst the broader population of fathers targeted by universal preventive parenting programs, which includes those whose children have never experienced mental health problems. Having insufficient time to commit to participating has been cited as a barrier to fathers’ engagement with parenting interventions [23,24,39], which is congruent with evidence suggesting that fathers spend more time in paid work and less time on child-rearing activities than mothers [40]. While there is some evidence to suggest that fathers may participate in a similar number of sessions to mothers once enrolled [41], low rates of initial enrolment across the parenting literature proscribe any clear consensus. It is possible that program length is a deterrent to those fathers who opt not to enrol in parenting interventions, whose views are under-researched. A clearer understanding of fathers’ preferred number of sessions can help guide development of programs that are of an acceptable length to fathers.

### 1.3. Program Benefits

Program benefits refer to improvements in parenting or the parent-child relationship that can be anticipated as a result of program participation. Salari and Filus [42] found that perceived benefits predicted both mothers’ and fathers’ intent to enrol in universally targeted parenting programs. However, the effects of specific benefits were not further investigated, and nor was the differential prediction of benefits for different program delivery formats (e.g., web-based compared to group, seminar, or individual face-to-face formats). Tully et al. [25] found that 16.2% of fathers of children aged between 2–16 years reported that not knowing what a parenting intervention is about was a barrier to participation, and the perception that parenting interventions are not relevant or of benefit has been identified as an engagement barrier for fathers [24]. Sicouri et al. [24] found that the inclusion of information in parenting interventions that is relevant, interesting, and to some extent father-specific, was a key preference held by fathers. Building a positive relationship with their child, increasing their child’s confidence and social skills, and understanding the importance of fathers in children’s development were rated as the most important parenting program topics in a cross-sectional survey of Australian fathers [23]. However, only small differences in topic ratings were observed as fathers made independent ratings of a list of topics, rather than choosing between topics. Since there is reason to believe that fathers’ understanding of the benefits associated with participating in web-based preventive parenting interventions may influence their choice to enrol, a clearer understanding of which benefits are most preferred by fathers can inform effective selection and marketing of program content to fathers.

### 1.4. Program Participants

Program participants describe members of the family who participate in the program. Fabiano et al. [43] found that fathers preferred preventive family interventions that included their partner, which contrasted with mothers who preferred not to participate with their partner. Both mothers and fathers preferred an intervention that involved their child, and no statistically significant differences were found between mothers and fathers in this preference [43]. Moreover, while a number of focus group studies have found that fathers express preferences for father-only group programs [23,24], a survey found that interventions which engage both parents were preferred by fathers over those where they were expected to attend alone [25]. Cowan et al. [44] found that in a face-to-face preventive intervention with low-income families, fathers’ engagement improved when mothers attended the first meeting. However, no studies have investigated how these preferences for involvement of parenting partners or children generalise to programs that used web-based delivery formats. Investigation of the influence of program participants on fathers’ choices is warranted to address inconsistencies in previous findings and investigate how they extend to web-based preventive programs.

### 1.5. User Control over Program Content

User control over program content refers to the extent to which the user has agency over the content that is included in an individualized program. A range of studies have highlighted fathers’ desire for flexibility and personalisation of program content to enhance their interest in participating in parenting interventions [31,39]. The perceived personal relevance of program content has been found to be highly valued by fathers [23,25]. While the content selection process was highly relevant to a segment of parents who preferred an ‘Information’ oriented delivery of interim resources while waitlisted for child mental health service, which included a higher proportion of fathers than other segments [38,45], this segment of parents were more likely to prefer content to be selected by a therapist over self-selecting content. This preference conflicts with the notion that the ability to retain perceived control is central to male help-seeking [46]. The ability to select personally relevant program content within web-based preventive programs would be consistent with user interface design principles of personalization, user control and flexibility to increase the appeal of computer-delivered programs [47]. While control over content selection has the potential to improve the adaptability of web-based preventive parenting programs for fathers, it is important to first understand whether the opportunity to personalise program content does influence their appeal for fathers in the context of other relevant program attributes.

### 1.6. Aims and Hypotheses

Understanding fathers’ preferences for web-based parenting programs can inform the development of father-inclusive parenting programs. Increasing paternal uptake of web-based parenting programs for adolescent mental health can potentially address parenting factors amongst fathers and consequently improve adolescent mental health outcomes. Therefore, the aims of this study were to use a DCE design to explore in a sample of fathers of adolescents aged 12–18 years, (a) the relative influence of *number of sessions*, *program benefits*, *program participants*, and *user control over program content* on their preferred web-based parenting program; and (b) whether selected father characteristics were associated with their preferences. We hypothesized that fathers would be more likely to prefer programs with fewer sessions, and that programs where fathers could choose program topics would be preferred over those where program topics were based on expert recommendation. We also hypothesized that program participants and program benefits would influence fathers’ preferences for web-based parenting programs, but as these attributes were exploratory, we did not form directional hypotheses.

## 2. Methods

### 2.1. Design

This study utilized a cross-sectional survey design. For the DCE, a full factorial design that included three attributes with three levels and one attribute with two levels was selected. In contrast with ‘efficient’ or ‘optimal main effect’ designs, full factorial designs give an equal probability of seeing every possible choice set. Given the known challenges inherent in recruiting fathers into research studies [20], we chose to reduce sampling requirements by using a binary discrete choice model in which respondents are presented with two parenting program options at a time. This model reduces the cognitive burden to participants for each choice set, and thereby increases the number of observations that can be collected from each respondent. Ethical approval was obtained from the Monash University Human Research Ethics Committee, Project ID 21846.

### 2.2. Recruitment

Participants were recruited through both community sampling and the use of paid survey panels identified through Prolific Academic and Qualtrics Panels. Prolific Academic (http://www.prolific.ac (last accessed on 12 May 2021)) is an online research recruitment platform that allows researchers to identify individuals who are interested in participating in online research studies, whilst Qualtrics Panels (https://www.qualtrics.com/au/research-services/online-sample/ (last accessed on 31 May 2021)) is a recruitment service that partners with online panel providers to identify research samples. Crowdsourcing platforms have been found to be an efficient source of recruitment for large samples of fathers, that can yield high quality and reliable data [48].

Convenience sampling was used to recruit participants to the community subsample (*n* = 49) by advertising through emailing lists of community organisations, and on social media sites such as Facebook, Twitter, Reddit and Instagram. Fathers were also recruited via word of mouth and snowballing. Advertisements described the project as a study of fathers’ preferences for online parenting programs for adolescent mental health. Eligible community members were invited to complete the survey by accessing an included link.

Participants on the Prolific Academic platform who had responded to pre-screening demographic questions that they were male, resided in Australia, and were a parent, were invited to respond to a brief screening questionnaire which asked the age of their children. One hundred and forty-one individuals responded to this screening questionnaire. Those whose response indicated they had at least one child between the ages of 12 and 18 years (*n* = 51) were then invited to respond to a 15-min survey. Of those eligible, 43 fathers completed the survey.

Qualtrics Panels participants were identified with support from the Qualtrics project coordinator, who was provided with the inclusion and exclusion criteria for the study. Qualtrics partners with a range of online panel providers to identify research samples. Respondents who are likely to qualify are randomly selected from double opt-in research panels, and then matched from their profile to their survey responses by their demographic information. Data quality is ensured by replacing responses that fail quality checks, which include duplications, non-differentiation in choices, suspicious open-text responses, or respondents who complete their survey in less than half of the median response time. Recaptcha and RelevantID are used to prevent fraudulent responses [49]. The majority of responses were collected by panels with ISO Certification 20252:2019, which specifies quality control processes including the requirement for respondents to provide a valid Australian bank account to receive incentives.

Calculation of minimum sample size requires researchers to determine initial beliefs about parameter values [50]. Due to the exploratory nature of this study, expected parameter values were determined by modelling a small subset of initially collected data (*n* = 20). These revealed that with an α error probability of 0.05, a minimum of 970 observations (*n* = 39, as each participant responded to 25 choice sets) was needed to obtain statistical power for main effects analyses at the 0.80 level recommended by Cohen [51].

### 2.3. Participants

Participants were 171 fathers of at least one adolescent child. To be eligible for this study, participants had to (a) identify as male, (b) be a caregiver to at least one adolescent aged 12–18 years, (c) reside in Australia, and (d) have sufficient English language proficiency to respond to a 15-min online survey. Demographic characteristics of the sample are detailed in Table 1, broken down by recruitment source.

Almost half of the combined sample (49.7%) rated their knowledge of adolescent mental health as ‘good’ or ‘excellent’. When asked about their confidence in their parenting for adolescent mental health, 31.0% of the sample indicated they were ‘moderately confident’ or ‘extremely confident’, and 56.1% were ‘moderately confident’ or ‘extremely confident’ using the internet to identify resources and information on different topics. After responding to demographic questions and choice sets, 91.2% indicated they would be interested in using an online program designed to help parents develop skills and knowledge to reduce their adolescent’s risk of mental health difficulties like those described in the survey, if given the choice. Chi-square tests showed differences across subsamples only in partner employment (χ^2^ = 20.53, *p* = 0.01), education (χ^2^ = 28.36, *p* = 0.01), and father lifetime mental health diagnosis (χ^2^ = 7.47, *p* = 0.03). A higher proportion of fathers recruited from Prolific Academic were tertiary educated and had partners that were employed full time. Fathers recruited from the community were more likely to report a lifetime mental health diagnosis.

### 2.4. Instruments

Data was collected with a cross-sectional survey hosted online by Qualtrics software [52]. Four blocks of questions were administered sequentially. The first block requested demographic information including age, country of birth, number of children, marital status, employment status, partner’s employment status, education, household income, and cultural background. The second block comprised questions on fathers’ prior use of parenting programs, perceived knowledge of adolescent mental health, confidence in parenting for adolescent mental health, confidence using the internet to identify resources and information, current or previous mental health diagnosis in themselves and in their adolescent, and whether the role of parenting for adolescent mental health was mostly theirs, mostly their partner’s, or shared equally. The third block comprised 25 randomised choice sets to determine preferences (additional details below). Finally, the fourth block included a question about whether the respondent would choose to use a program like those described in the survey, if offered the opportunity. Those who indicated ‘no’ were invited to provide further information as to their reasons for this preference.

**Figure 1:**
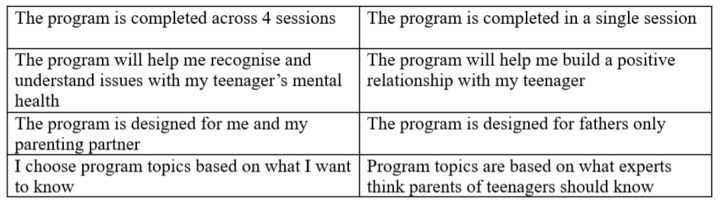
DCE scenario and example choice set.

The survey requested participants to repeatedly choose between pairs of hypothetical web-based parenting programs. Each hypothetical program consisted of four attributes, with descriptors of various alternatives (known as levels) for each attribute. The attribute *number of sessions* had three levels which included 1 session, 4 sessions, or 8 sessions. The attribute *program benefits* (i.e., benefits conferred on the parent by participating in the program) comprised three levels which included recognizing and understanding issues with their adolescent’s mental health, preventing or assisting with issues with their adolescent’s mental health, or building a positive relationship with their adolescent. The attribute *program participants* (i.e., members of the family the program is designed to involve) had three levels which included fathers only, me and my parenting partner, and me and my adolescent. Finally, the attribute *user control over program content* had two levels, which included the user choosing program topics based on their preferences, and the user being allocated program topics based on expert recommendation. The DCE was presented in the format shown in Figure 1.

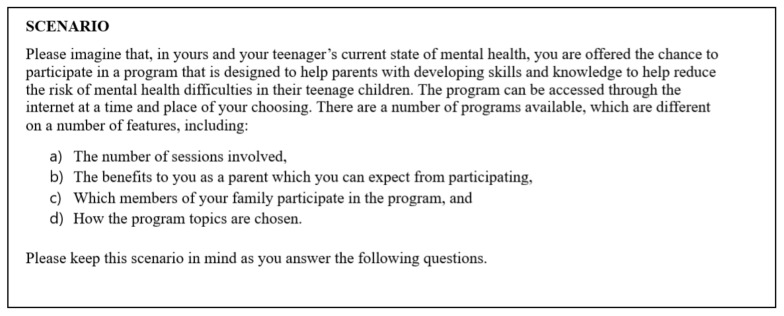



Example choice set. Participants were asked to indicate which option they would select if given the choice between the two programs.

### 2.5. Development of Discrete Choice Experiment

In this study we combined literature reviews and qualitative research to inform attribute generation. Consistent with recommendations by Louviere et al. [36] and Ryan et al. [53], an initial literature review was conducted of relevant studies to identify conceptual attributes relevant to web-based parenting programs within published and grey literature. Concurrently, qualitative data was collected through semi-structured interviews to support attribute development [37]. Seventeen conceptual attributes were identified from literature searches and qualitative data. These were rated by the first author (a doctoral research student in Clinical Psychology) against an attribute development framework with nine selection criteria, modified from Helter and Boehler [35] and Coast et al. [54]. The remaining conceptual attributes and corresponding levels were further reduced and refined through review and discussion with the research team, including researchers with expertise in DCE design and parenting program development, until consensus was reached on the content and format of included items.

All attributes and levels were randomized in a full-factorial design, producing 54 unique parenting program options. These were combined in all possible pairings to construct 1431 (*N*(*N* − 1)/2) ‘choice sets’ which each comprised two hypothetical parenting programs for comparison. A random subset of 25 choice sets was drawn for each individual survey. The number of choices sets was selected based on acceptable respondent burden, identified through survey piloting and consultation with the research team. The left/right orientation of parenting program options were randomized within choice sets when presented on screen (top/bottom for surveys that were completed using a smartphone).

## 3. Procedure

### 3.1. Piloting

Pre-testing DCE surveys through piloting is recommended to identify the optimal number of choice sets to be presented, appropriate survey length, and readability [55]. Piloting was undertaken with four research assistants, two researchers with expertise in parenting program development, and four fathers who had participated in the qualitative research study.

### 3.2. Data Collection

Data collection was initiated shortly after piloting was completed and occurred from June 2020 to May 2021. Before commencing the survey, respondents from all recruitment streams were asked to read the participant information and provide their consent. Participants were fully informed about the purpose and nature of the research, including any incentives that were offered for their participation. If consent was not provided, the survey then terminated. Those who provided consent and met eligibility criteria continued to the demographics questionnaire and DCE survey.

### 3.3. Data Analysis

Data were analysed in R version 3.4.1 using packages tidyverse and ordinal to process data and produce cumulative link models [56,57]. Cumulative link models assume the presence of an underlying continuous latent variable with cumulative distribution function. They observe the boundaries of the response variable by treating it as nominal, allow different rates of change across levels of independent variables, and account for correlations between responses by the same individual. A probit link function was used, corresponding to a Gaussian assumption of a random utility model of preferences. This does not imply that any observed data are normal, but instead that internal psychological preference strengths are normally distributed. This is a standard assumption used in most random utility models.

Summary statistics were produced for demographic variables, and inferences regarding the influence of program attributes on choice of program were analysed using cumulative link models to predict choices, using attribute levels as predictors. *User control over program content*, *program benefits* and *program participants* attributes were modeled as ordinal variables, with one level of each specified as the reference category. *Number of sessions* was modeled as a continuous variable, allowing for time equivalence for moving between levels. The relative influence of different attributes on choice was quantified using the standardized coefficients produced by these models (expressed as z-scores, otherwise known as beta-coefficients).

To address subsidiary research questions about whether father characteristics are associated with their preferences for program attributes, we included predetermined variables of interest as predictors in this model: whether fathers reported prior use of a parenting program, were tertiary educated, reported a past or current mental health diagnosis in themselves or their adolescent, or were part of a dual-working family. Prior use of a parenting program, tertiary education, past or current mental health diagnosis for fathers and their adolescent, and being part of a dual working family were computed as binary variables. Dual working families were defined as those in which both the participant and their partner were engaged in part- or full-time work. Confidence in parenting for adolescent mental health was treated as a continuous predictor and measured using a 5-point Likert scale ranging from ‘Not at all confident’ to ‘Extremely confident’. We also examined whether fathers’ perceived role of parenting for adolescent mental health issues (mostly themselves vs. mostly their partner vs. shared equally) and confidence in parenting for adolescent mental health interacted with their preferences.

## 4. Results

**Figure 2:**
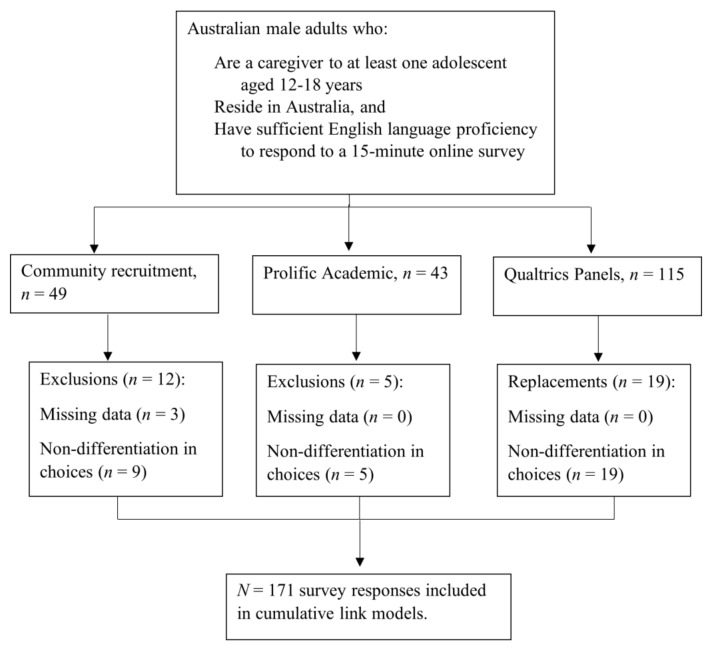
Participant flow and reasons for exclusion.

Prior to main analyses, data was screened for missing values, left/right selection bias, and non-differentiation of choices. Three cases with <80% of data were deemed unsuitable for analysis and excluded, as were 33 cases with evidence of non-differentiation of choices. Figure 2 shows participant flow by recruitment source and reasons for exclusion.

As there were many different levels of possible complexity in the model (corresponding to including more and higher-level interaction effects), we initially reviewed the most complex model with all two-, three-, and four- way interactions and identified a significant interaction effect between *number of sessions* and *program participants*. Fathers who were more likely to prefer to complete the program with their partner were less likely to prefer programs with fewer sessions (*z* = 2.79, *p* < 0.001). No other significant interactions were found between attribute levels. The Akaike information criterion index (AIC) for the model which accounted for this interaction was marginally better than the AIC for the main-effects-only cumulative link model (5768.49 vs. 5777.28), and the AIC for the most complex model (5814.35). Hence here we report the model that includes only the interaction between numbers of sessions and program participants (see Supplemental Table S1 for full model).

### 4.1. Influence of Program Attributes on Fathers’ Preferences

Fathers’ choices were most strongly influenced by *program participants*. Fathers were more likely to prefer a program that they could complete with their adolescent (*z* = 10.06, *p* < 0.0001) or parenting partner (*z* = 7.30, *p* < 0.0001), than a program that was designed for fathers only. Fathers’ choices were also influenced by the *user control over program content*. Fathers were more likely to prefer a program where program topics were allocated based on expert recommendation (*z* = −4.31, *p* < 0.0001), than a program where users choose program topics based on their own preferences. *Number of sessions* also influenced fathers’ choices (*z* = −2.94, *p* < 0.001). Fathers were more likely to prefer a program with 4 sessions to a program with 8 sessions. They also preferred programs with 1 session to a program with 8 sessions.

**Figure 3:**
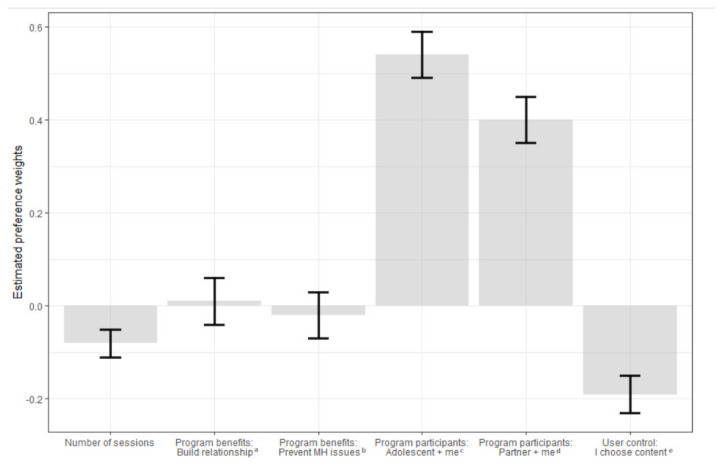
Estimated preference weights for attribute levels. *Note*. MH, mental health. Referent level for program benefits = ‘recognising and understanding issues with their adolescent’s mental health’. Referent level for program participants = ‘a program designed for fathers only’. Referent level for user control = ‘the user is allocated program topics based on expert recommendation’. ^a^ Building a positive relationship with their adolescent. ^b^ Preventing or assisting with issues with their adolescent’s mental health. ^c^ Program is designed to involve fathers and their parenting partner. ^d^ Program is designed to involve fathers and their adolescent child. ^e^ User chooses program content based on their preferences.

In contrast, *program benefits* did not have a statistically significant effect on fathers’ choices (prevent adolescent experiencing mental health issues, *p* = 0.70; build a positive relationship with my adolescent, *p* = 0.83). Figure 3 shows the strength of preference for each attribute level in comparison to a reference category, as represented by beta values.

### 4.2. Influence of Father Characteristics on Preferred Program Attributes

We tested exploratory associations between selected demographic variables and program attributes by sequentially entering interaction terms between program attributes and each demographic variable of interest, and deleting those where no association was found. We report a final model with those associations here (see Supplemental Table S2 for full model). Fathers’ preferences for *program participants* did not differ by whether they were in a dual-working family, the presence of a past or current mental health diagnosis in themselves or their adolescent child, or confidence in parenting for adolescent mental health. However, compared to those who had previously used a parenting program, fathers who had not previously used a parenting program had a stronger preference for a program they could complete with their partner, over a program designed for fathers only (*z* = 2.92, *p* = 0.01). Additionally, compared to those with a tertiary qualification, non-tertiary educated fathers had a less strong preference for a program designed for them to complete with their adolescent over a program designed for fathers only (*z* = −2.93, *p* = 0.01). Fathers who believed the role of parenting for adolescent mental health was shared equally by them and their parenting partner had a stronger preference for a program they could complete with their partner (*z* = 4.58, *p* < 0.0001) or adolescent child (*z* = 4.28, *p* < 0.0001) than those who believed it was mostly theirs or their partner’s role.

Fathers’ preferences for *number of sessions* did not differ by prior use of a parenting program, education, perceived role of parenting for adolescent mental health, level of confidence in parenting for adolescent mental health or presence of a past or current mental health diagnosis in themselves or their adolescent child. Fathers in dual-working families had a less strong preference for a program with 8 sessions, and stronger preference for a program with 1 or 4 sessions, than those from families in which either they or their partner were not employed in any capacity (*z* = 2.35, *p* = 0.02).

Finally, fathers’ preferences for *program benefits* and *user control over program content* did not differ by any of the selected participant characteristics.

## 5. Discussion

This study explored whether fathers’ preferences for web-based parenting programs for adolescent mental health were influenced by *number of sessions*, *program benefits*, *program participants*, and *user control over program content*. We also sought to investigate whether these preferences were associated with fathers’ prior use of a parenting program, education, history of mental health diagnosis in themselves or their adolescent, employment, perceived role of parenting for adolescent mental health issues, and confidence in parenting for adolescent mental health.

We found that fathers’ preferences for web-based parenting programs were most strongly influenced by the family members the program is designed to involve. Specifically, fathers were more likely to prefer a program that they could complete with their adolescent child or parenting partner over one that was designed for fathers only. This finding suggests that father-inclusive approaches to program development which target fathers alone may have less broad appeal than strategies that seek to incentivise fathers’ participation alongside their parenting partner or adolescent child. Evidence regarding fathers’ preferences for father-only parenting programs is inconsistent in the existing literature. Although some studies have found that fathers report a preference for father-only group parenting programs [23,24], survey-based research has shown that fathers rate both single-session and weekly interventions that engage both parents as more preferred than father-only interventions [25]. Furthermore, co-parent or family programs have reported better child and parent outcomes than those that only involve one parent from two-parent families [31,44,58]. It is possible that self-selection bias accounted for fathers’ expressed preferences for father-only group programs in previous qualitative studies. Fathers who volunteer to participate in focus groups on parenting interventions may have higher mental health literacy and be more willing to engage in a group-based delivery format than the general population. Further, Frank et al. [23] compared fathers’ preference ratings for father-only group programs to web-based delivery or couples group programs (alongside other possible delivery formats), making it difficult to discern whether preference ratings related to family members participating in the program, web-based delivery, or participating within groups. Fathers were not asked to choose between programs but instead assigned ratings to each possible delivery format, which may have inflated preference ratings for each delivery format. In the current study, fathers preferred a program that included their adolescent child above both other options.

Fathers’ preferences for inclusion of their child in parenting interventions has been given limited attention in the extant literature. However, this finding does support the strong importance placed by fathers on using parenting programs for building positive relationships with their child [23]. Fathers have previously reported that encouragement from their partner to attend is the strongest motivational factor for engagement [59], and generally this supports the idea that inviting father involvement through whole-family participation may optimise father engagement. Although fathers’ preference for program participants did not differ according to whether they were in a dual-working family, presence of a past or current mental health diagnosis for themselves or their adolescent child, or confidence in parenting for adolescent mental health, further exploration showed that tertiary-educated fathers had a stronger preference for programs that included their adolescent child. Parent educational attainment is a well-established predictor of parenting program enrolment and attendance [17,60,61]. While Fleming et al. [62] found that parents were more likely to attend one or more sessions of a parenting program adapted with two sessions that included their adolescent child than a standard, parent-only version, educational attainment did not differentially predict enrolment across versions of the program. Moreover, although 84% of Fleming et al.’s [62] sample were female, a study of paternal engagement in a family-focused intervention showed that paternal education did not predict father participation [63]. In the current study we distinguished between parents who were tertiary and non-tertiary educated rather than decomposing these categories into ranked levels of education. It is possible that the distinction between tertiary and non-tertiary educated parents more clearly delineates engagement behaviors and parent preferences, and although this preference was more prominent in the tertiary-educated group, both groups did prefer programs which included their adolescent child to those designed for fathers only. Additionally, in this study fathers who had not previously used a parenting program had stronger preferences for programs they could complete with their parenting partner, and fathers who believed the role of parenting for adolescent mental health was shared equally by them and their parenting partner indicated stronger preferences both for programs that included their adolescent and programs that included their parenting partner. Perspectives of fathers who have never used parenting programs are under-investigated, and these findings suggest that fathers with less experience using parenting programs and who believe they play a shared role in parenting for adolescent mental health are more likely to use programs that they can participate in with other family members.

Fathers were also more likely to prefer a program in which content was predetermined for them by subject matter experts over a program where users could select content based on their own preferences. This did not differ according to the selected father characteristics tested in our model. Contrary to what was hypothesized, this suggests that control over content selection may be less of a priority to fathers than confidence in the expert knowledge underpinning program topics. In prior research, fathers have rated ‘demonstrated effectiveness’ as most influential on their decision to participate in a parenting program among a range of program features [23], and have expressed a clear preference for evidence-based interventions [64]. Alternately, the current finding may reflect previous consistent findings that fathers report limited awareness of parenting programs [24,25,65], with one study reporting that only 13% of fathers surveyed had heard of available parenting programs [23]. Limited understanding of what is involved in parenting interventions has been cited as a barrier to paternal participation [25]. Thus, the finding that fathers may prefer program content that is predetermined at the cost of personal selection may reflect a lack of confidence in their capacity to choose content.

Regarding number of sessions, we found that fathers were most likely to prefer a program with four sessions compared to a program with one or eight sessions, with eight sessions being their least preferred option. Fathers in dual-working families and fathers whose adolescent child has had a past or current mental health diagnosis reported a less strong preference for four sessions or one session over eight sessions. However, fathers’ preferences did not differ according to prior use of parenting programs, presence of past or current mental health diagnosis in themselves, perceived role of parenting for adolescent mental health issues, or confidence in parenting for adolescent mental health. Higher adherence—observed program usage, proportionate to the amount intended to be therapeutic by program developers—to parenting programs is commonly associated with improved outcomes [66,67], which is often attributed to the importance of dosage, or the number of sessions completed. This is of concern, as systematic reviews of ongoing engagement in preventive parenting programs indicate that on average parents actually attend between 3 and 7 sessions, or in the range of 40.6–87.5% of intended modules for technology-assisted parenting programs [68,69], which was consistent with fathers’ preference for a program comprising four sessions. An alternative explanation, however, is that the benefits of a program may be conferred more by receiving an adequate proportion of active intervention components than they are by completing a higher number of sessions [70]. If this is the case, increasing the density of active intervention components within a program may allow the time commitment to be reduced and improve adherence rates, whilst maintaining the maximal benefits conferred by the program.

Finally, benefits conferred by participating in a program did not have a statistically significant effect on fathers’ preferences. Perceived benefits have been shown to positively predict parenting program attendance [60], but the study did not differentiate between possible types of benefits and predominantly sampled mothers (93%). Frank et al. [23] report that Australian fathers rated a range of parenting program topics as highly important, but there was little distinction found in importance ratings across topics. When asked to trade off preferred program topics against other attributes in this experiment, including number of sessions, program participants, and user control over program content, fathers prioritized the latter aspects of program delivery. Fathers may still value the inclusion of relevant benefits conferred by participating in programs, but in terms of influencing engagement choices, other attributes tested in this experiment are likely to have a stronger impact on fathers’ decision making.

## 6. Limitations and Future Directions

Although external validity for DCEs is generally high, with stated preferences often corresponding well to real-life choices [71], it is possible that fathers may make different choices when faced with real-world parenting programs than the hypothetical programs presented in this experiment. This is a limitation of all data collection methods that rely on stated preferences (including qualitative interviews and other self-report questionnaires) to elicit fathers’ preferences for web-based preventive parenting programs. As fathers’ actual parenting program choices have not yet been compared with their stated preferences, further investigation is needed into how well stated preference data predicts actual behavior in this domain. For example, a future study could implement program adaptations that align with fathers’ stated preferences in this study, such as a module that can be completed together with their adolescent child, and compare the uptake of the adapted versus the original program amongst fathers.

Our study utilized a relatively small sample in comparison to other DCEs of parenting interventions [38,43,45] and broader healthcare services [55,72,73]. To offset this, we limited the number of attributes used to describe hypothetical programs. This allowed us to reduce the cognitive burden of each choice set and reduce sampling requirements for a full-factorial design. Attribute levels were identified using preliminary qualitative research and input from the research team, corresponding to realistic and manipulable aspects of parenting program development. The interpretation of the strength of predictive importance for each attribute may change if more or differently defined levels were included, or if other potential parenting program features such as length of sessions, program cost, or aspects of delivery format were included. In order to ensure that fathers responded meaningfully to a sufficient number of choice sets, we did not include an opt-out option with each choice set. In a real-world scenario, fathers would have the option not to use a parenting program of any kind. However, in this sample only 8.8% of fathers indicated that they would not be interested in using a parenting program like those described if offered the opportunity, suggesting that respondents are likely to have traded-off attributes accurately.

## 7. Conclusions

This is one of few early studies to provide an in-depth examination of engagement factors for parenting programs amongst fathers of adolescents, and to our knowledge is the first to utilise a DCE methodology to explore fathers’ preferences for features of web-based preventive parenting programs. Fathers in this study preferred programs that they could complete alongside their parenting partner or adolescent child, that were delivered across fewer sessions, and where program content was selected for them based on expert recommendation. Benefits conferred on fathers through program use did not have a statistically significant effect on their choices when weighed against the other program characteristics. Program developers should take fathers’ preferences for aspects of program delivery into account when designing web-based parenting programs for adolescent mental health or modifying them to increase father-inclusiveness. Improving father engagement by increasing the appeal of web-based parenting programs has the potential to improve parenting and adolescent mental health outcomes at a population level.

## Figures and Tables

**Table 1:** Participant characteristics.

Characteristics	Combined Sample (*n* = 171)		Community Subsample (*n* = 37)		Prolific Subsample (*n* = 38)		Qualtrics Subsample (*n* = 96)	
	*n*	%	*n*	*%*	*n*	%	*n*	%
Age								
25–34	14	8.2	0	0.0	7	18.5	7	7.3
35–44	49	28.7	8	21.6	9	23.7	32	33.3
45–54	80	46.8	24	64.8	18	47.4	38	39.6
55–64	25	14.6	5	13.5	4	10.5	16	16.7
≥65+	3	1.8	0	0.0	0	0.0	3	3.1
Highest education qualification								
Secondary	31	18.0	9	24.3	4	10.3	18	18.8
Apprenticeship	10	5.8	2	5.4	2	5.3	6	6.3
TAFE certificate/other technical qualification	30	17.5	2	5.4	5	13.2	23	24.0
Undergraduate degree	50	29.2	9	24.3	18	47.4	23	24.0
Postgraduate degree	50	29.2	15	40.5	9	23.7	26	27.1
Country of birth								
Australia	143	83.6	31	83.8	30	78.9	82	85.4
United Kingdom	7	4.1	3	8.1	2	5.3	2	2.1
United States of America	3	1.8	0	0.0	1	2.6	2	2.1
Malaysia	2	1.2	1	2.7	0	0.0	1	1.0
New Zealand	2	1.2	1	2.7	0	0.0	1	1.0
China	2	1.2	0	0.0	1	2.6	1	1.0
France	1	0.6	0	0.0	1	2.6	0	0.0
Germany	1	0.6	0	0.0	0	0.0	1	1.0
Hong Kong	1	0.6	0	0.0	1	2.6	0	0.0
Ireland	1	0.6	1	2.7	0	0.0	0	0.0
Pakistan	1	0.6	0	0.0	1	2.6	0	0.0
Papua New Guinea	1	0.6	0	0.0	0	0.0	1	1.0
Russia	1	0.6	0	0.0	1	2.6	0	0.0
Slovenia	1	0.6	0	0.0	0	0.0	1	1.0
Sudan	1	0.6	0	0.0	0	0.0	1	1.0
Taiwan	1	0.6	0	0.0	0	0.0	1	1.0
Uruguay	1	0.6	0	0.0	0	0.0	1	1.0
Zimbabwe	1	0.6	0	0.0	0	0.0	1	1.0
Household income (AUD)								
<$40,000	11	6.4	3	8.1	2	5.3	6	6.3
$40,000–$79,999	24	14.0	2	5.4	6	15.8	16	16.7
$80,000–$119,999	35	20.5	5	13.5	10	26.3	20	20.8
$120,000–$159,000	41	24.0	11	29.7	7	18.4	23	24.0
$160,000–$199,000	19	11.1	3	8.1	2	5.3	14	14.6
$200,000–$239,000	20	11.7	9	24.3	6	15.8	5	5.2
$240,000–$279,999	9	5.3	1	2.7	2	5.3	5	5.2
$280,000–$319,000	3	1.8	1	2.7	0	0.0	2	2.1
$320,000 or more	9	5.3	2	5.4	2	5.3	5	5.2
Number of children								
2	40	23.4	5	13.5	8	21.1	27	28.1
3	76	44.4	19	51.4	14	36.8	43	44.8
4	36	21.1	9	24.3	12	31.6	15	15.6
5	16	9.4	4	10.8	4	10.5	8	8.3
6	3	1.8	0	0.0	0	0.0	3	3.1
Marital status								
Married/Domestic partnership	141	82.5	27	73.0	31	81.6	83	86.5
Divorced	14	8.2	6	16.2	4	10.5	4	4.2
Separated	9	5.3	3	8.1	0	0.0	6	6.3
Single (never partnered)	5	2.9	0	0.0	3	7.9	2	2.1
Widowed	1	0.6	0	0.0	0	0.0	1	1.0
Employment								
Full time	123	71.9	28	75.7	24	63.2	71	74.0
Part time	16	9.4	2	5.4	5	13.2	9	9.4
Self-employed	17	9.9	5	13.5	6	15.8	6	6.3
Not employed/unable to work	9	5.3	2	5.4	1	2.6	6	6.3
Homemaker	4	2.3	0	0.0	0	0.0	4	4.2
Partner employment								
Full time	68	39.8	14	37.8	19	50.0	35	36.5
Part time	33	19.3	9	24.3	6	15.8	18	18.8
Self-employed	8	4.7	4	10.8	2	5.3	2	2.1
Not employed/ unable to work	4	2.3	0	0.0	0	0.0	4	4.2
Homemaker	27	15.8	0	0.0	4	10.5	23	24.0
Previous program use	44	25.7	11	29.7	9	23.7	24	25.0
Role of parenting for adolescent mental health								
Mostly me	34	19.9	4	10.8	6	15.8	24	25.0
Mostly my partner	23	13.5	5	13.5	5	13.2	13	13.5
Equally me and my partner	114	66.7	28	75.7	27	71.1	59	61.5
Adolescent mental health diagnosis (current)	48	28.1	9	24.3	13	34.2	26	27.1
Adolescent mental health diagnosis (lifetime)	59	34.5	15	40.5	13	34.2	31	32.3
Father mental health diagnosis (current)	33	19.3	11	29.7	5	13.2	17	17.7
Father mental health diagnosis (lifetime)	59	34.5	20	54.1	11	28.9	28	29.2

Note: TAFE, Technical and Further Education. AUD, Australian dollar.

## Supplementary Materials

The following are available online at https://www.mdpi.com/article/10.3390/ijerph182312340/s1, Table S1: Predictors of father program choice, Table S2: Influence of father demographic characteristics on predictors of program choice.
