# Supplementary material for: Enhancing Engagement of Fathers in Web-Based Preventive Parenting Programs for Adolescent Mental Health: A Discrete Choice Experiment
Source: Int J Environ Res Public Health. 2021 Nov 24;18(23):12340. doi: 10.3390/ijerph182312340 (PMC8656658; doi:10.3390/ijerph182312340)
Supplement: Supplementary file 1 [file ijerph-18-12340-s001.zip › ijerph-1455504-supplementary.pdf]

Supplemental Materials

Supplemental Table S1. *Predictors of father program choice.*

	β (95% CI)	*p*
Number of sessions	-0.08 (-0.11, -0.05)	< .01
Program benefits		
Build relationship with adolescent	0.01 (-0.04, 0.06)	.83
Prevent adolescent experiencing mental health issues	-0.02 (-0.07, 0.03)	.70
Program participants		
Parenting partner	0.39 (0.34, 0.44)	< .001
Adolescent child	0.54 (0.49, 0.59)	< .001
User control	-0.19 (-0.23, -0.15)	< .01
Number of sessions x Program participants: parenting partner	0.13 (0.09, 0.17)	< .001

Supplemental Table S2. *Influence of father demographic characteristics on predictors of program choice.*

	β (95% CI)	*p*
Number of sessions	-0.09 (-0.25, 0.09)	.61
Program benefits		
Build relationship with adolescent	-0.37 (-0.58, -0.15)	.09
Prevent adolescent experiencing mental health issues	0.03 (-0.03, 0.09)	.66
Program participants		
Parenting partner	-1.39 (-1.76, -1.02)	< .001
Adolescent child	-0.06 (-0.43, 0.31)	.86
User control	-0.19 (-0.24, -0.14)	< .001
Number of sessions x Program participants: parenting partner	0.15 (0.10, 0.20)	< .01
Number of sessions x Dual working family	0.14 (0.07, 0.21)	.04
Number of sessions x Adolescent mental health diagnosis	-0.09 (-0.17, -0.01)	.27
Number of sessions x Father mental health diagnosis	-0.03 (-0.11, 0.05)	.69
Program benefits: build x Father mental health diagnosis	0.21 (0.09, 0.33)	.09
Program participants: partners x Prior program use	0.39 (0.26, 0.52)	< .01
Program participants: partners x Role of parenting for adolescent mental health	0.43 (0.33, 0.53)	< .001
Program participants: partners x Dual working family	0.26 (0.13, 0.39)	.05
Program participants: partners x Adolescent mental health diagnosis	-0.08 (-0.25, 0.09)	.65
Program participants: partners x Father mental health diagnosis	-0.02 (-0.16, 0.12)	.87
Program participants: teens x Education	-0.34 (-0.45, -0.23)	< .01
Program participants: teens x Role of parenting for adolescent mental health	0.40 (0.31, 0.49)	< .001
Program participants: teens x Adolescent mental health diagnosis	0.10 (-0.04, 0.24)	.51
